# 

**Published:** 1895-08

**Authors:** 


### PERSONAL.

Dr. M. A. Piche, veterinarian of the 1st Cavalry, has resigned his position to enter private practice as assistant to Dr. O. Bruneau, Montreal, Canada.

Dr. J. H. Going, M.R.C.V.S., formerly of the 7th Cavalry, more recently State Veterinarian of Kansas, 1889–92, succeeds Dr. Piche in the 1st Cavalry.

Veterinarian Neif, of California, appeared before the third annual Sanitary Convention of that State in June with a paper entitled, "The Rôle of the Veterinarian in Human Prophylactic Medicine."

President Stewart's address before the Missouri Valley Veterinary Association in 1894 contains so many good thoughts specially applicable to the conditions existing to-day that we feel no apology is needed for its publication at this time.

Professor W. L. Zuill reports a very gratifying sale of his translation of Friedberger and Frohner's *Pathology and Therapeutics of the Domestic Animals*, and its adoption as a text-book by the American, Canadian, and English veterianary colleges, with few exceptions. Such a reception as this gives strong promise of added literature to our libraries.

Dr. John Robertson, D.V.S. (Montreal), has obtained the rank of Sergeant in Troop "C," 2d U. S. Cavalry, having resigned as Veterinary Surgeon of that regiment. His first examination will be made this fall ; the second one a year hence.

### Marriages Among the Veterinarians.

Dr. John W. Gadsden, of Philadelphia, to Mrs. Lucy M. Michener, of Washington, D. C., on Thursday, July 11, 1895, at the Church of the Incarnation, Washington, D. C.

